# Progress Toward Poliomyelitis Eradication — Afghanistan, January 2012–September 2013

**Published:** 2013-11-22

**Authors:** World Health Organization (WHO) Country Office, Kabul, Afghanistan, Polio Eradication Dept, WHO Eastern Mediterranean Regional Office, Cairo, Egypt, Polio Eradication Dept, WHO, Geneva, Switzerland, Div of Viral Diseases, National Center for Immunization and Respiratory Diseases, Global Immunization Div, Center for Global Health, CDC

Since 2012, transmission of indigenous wild poliovirus (WPV) has been limited to three countries: Afghanistan, Pakistan, and Nigeria (1). This report describes polio eradication activities and progress in Afghanistan during January 2012–September 2013 and updates previous reports (2,3). During 2012, 37 WPV type 1 (WPV1) cases were confirmed in Afghanistan, compared with 80 cases in 2011; nine WPV1 cases were confirmed during January–September, 2013, compared with 26 WPV1 cases during the same period in 2012. Since November 2012, no WPV1 cases have been reported from the Southern Region, previously the main WPV reservoir in Afghanistan; all nine polio cases in 2013 were in the Eastern Region and caused by WPV1 that originated in Pakistan.* From October 2012 to March 2013, 14 polio cases caused by circulating vaccine-derived poliovirus type 2 (cVDPV2) were detected in the Southern Region.† During 2012–2013, strategies to improve supplemental immunization activity (SIA)§ effectiveness in 11 low-performing districts (LPDs)¶ in the Southern Region included increasing staff and supervisory training, implementing short-interval-additional-dose (SIAD) campaigns,** placing transit vaccination teams at the borders of districts inaccessible because of insecurity, and establishing permanent polio vaccination teams to vaccinate children quarterly. From March 2012 to August 2013, the percentage of children unreached during SIAs declined by 43% in the Southern Region but increased by 122% in the Eastern Region. Despite ongoing challenges, the government of Afghanistan continues to expand the application of innovative solutions to reach unvaccinated children in accessible and inaccessible districts.

## Immunization Activities

Children aged <1 year are recommended to receive 3 doses of trivalent oral poliovirus vaccine (tOPV) through routine immunization services. The estimated national coverage with 3 tOPV doses (OPV3) at age 1 year was 71% in 2012, compared with 68% in 2011 (4). OPV coverage through routine immunization services reported among children aged 6–23 months with nonpolio acute flaccid paralysis (NPAFP)†† is used as a proxy indicator for OPV3 coverage nationally and was 61% in 2011 and 62% in 2012, with considerable regional variability in 2012: Central, 87%; Badakhshan, 82%; Eastern, 79%; Northern, 75%; Western, 71%; Northeastern, 69%; Southeastern, 34%; and Southern, 15%.

**FIGURE 1:**
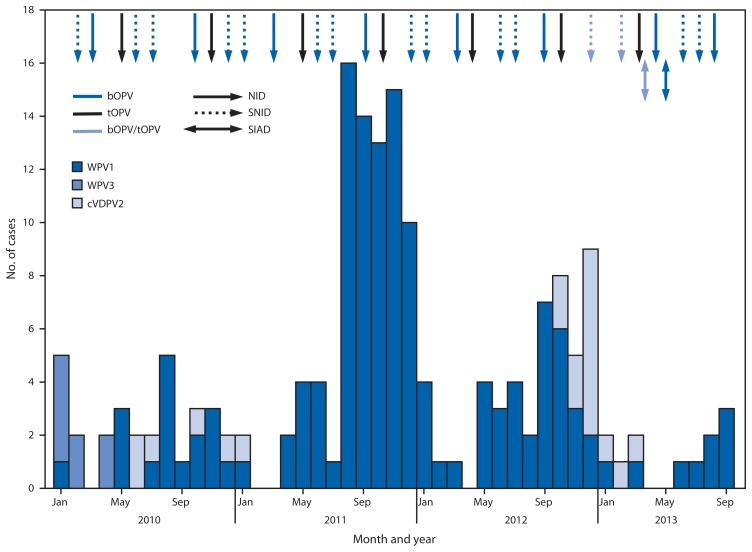
Number of cases of wild poliovirus types 1 (WPV1) and 3 (WPV3) and circulating vaccine-derived poliovirus type 2 (cVPDV2), type of supplementary immunization activity conducted, and type of vaccine used, by month — Afghanistan, 2010–2013* **Abbreviations:** NID = national immunization days; SNID = subnational immunization days; SIAD = short-interval-additional-dose campaign; bOPV = bivalent oral poliovirus vaccine; tOPV = trivalent oral poliovirus vaccine. * Data as of November 4, 2013.

All children aged <5 years are targeted to receive OPV through SIAs. During January 2012–September 2013, 14 SIAs were conducted, including seven national and seven subnational SIAs. Of these, nine SIAs used bivalent (types 1 and 3) OPV (bOPV), three used tOPV, and two used a combination of tOPV and bOPV (Figure 1). National SIAs targeted an estimated 8.3 million children aged <5 years. Subnational SIAs targeted an estimated 3.2 million children aged <5 years, primarily in the Eastern, Southeastern, Southern, and Western Regions. In 2013 to date, two SIAD rounds were implemented in the Southern and Eastern regions, targeting approximately 800,000 children aged <5 years with bOPV 1–2 weeks after the March and May SIAs.

The impact of SIAs in reaching children is monitored through post-SIA coverage assessment surveys in accessible areas, which are used to estimate the number of children missed because of programmatic issues, such as weak team performance or noncompliance of caretakers. The number of children unreached because of insecurity is estimated by using the target population in inaccessible areas.§§ During the first national SIA covered by this report, conducted in March 2012, an estimated 660,389 (9%) of 7,517,279 eligible children were unreached; of these, 331,824 (50%) were in the Southern Region, and 35,847 (5%) were in the Eastern Region.

In the Southern Region, during the March 2012 national SIA, the estimated number of unreached children was 331,824 (24%) of the 1,380,127 regional target population (303,402 [22%] missed in accessible areas and 28,422 [2%] because of insecurity, respectively). During the last national SIA covered by this report, in August 2013, an estimated 190,044 (13%) of the 1,434,833 regional target population were unreached (176,952 [12%] and 13,092 [1%], respectively, in accessible and inaccessible areas), representing a 43% decline from March 2012, because of improvements in reaching inaccessible children after negotiations with local leaders over access and in reducing the number of children missed for programmatic reasons.

In the Eastern Region, during the March 2012 national SIA, the estimated number of unreached children was 35,847 (5%) of the 748,285 regional target population (20,250 [3%] missed in accessible areas and 15,597 [2%] in areas inaccessible because of insecurity, respectively). During the August 2013 national SIA, an estimated 79,741 (10%) of the 834,944 regional target population were unreached (62,738 [8%] and 17,003 [2%] in accessible and inaccessible areas, respectively), an increase of 122% from March 2012, predominantly because of a threefold increase in children missed for programmatic reasons.

As determined by postcampaign coverage surveys, the quality of SIA implementation in the field remained the major challenge: approximately 50% of children were missed because they were not available at time of the vaccination team’s visit, and in most areas, the policy to revisit households with previously absent children was not implemented correctly. Nearly 25% of children were missed because caretakers reported that no vaccination team came to their house, suggesting weak SIA planning and supervision in these areas.

The proportion of children aged 6–23 months with NPAFP who have never received OPV (“zero-dose children”) is used as a combined proxy measure of the quality of routine and supplementary vaccination. In the Southern Region, the proportion of zero-dose children was 19% in 2011, 14% in 2012, and 3% in 2013. In the Eastern Region, the proportion of zero-dose children was 0% in 2011, 1% in 2012, and 8% in 2013. In the rest of the country, where accessibility is not a major problem, the proportions of zero-dose children were 1%, 2%, and 0% in 2011, 2012, and 2013, respectively.

## Acute Flaccid Paralysis (AFP) Surveillance

The indicators used to monitor the quality of AFP surveillance have been defined previously (5).¶¶ In 2012, the annual NPAFP rate was 9.5 per 100,000 population aged <15 years nationally (range among the eight regions: 6.7–12.1). In 2012, adequate specimens were collected for 92% of AFP cases nationally (range: 82%–98%) (Table).

## WPV and Vaccine-Derived Poliovirus (VDPV) Epidemiology

**FIGURE 2:**
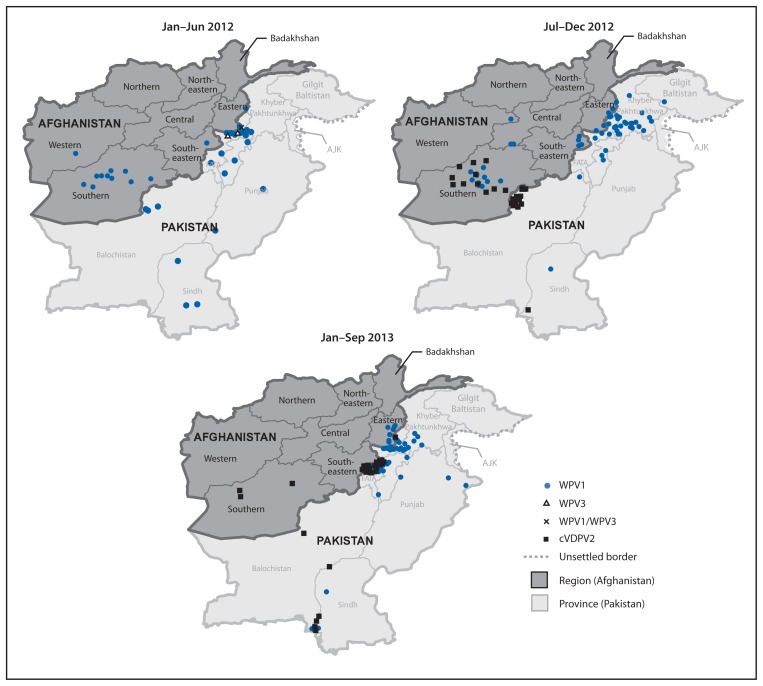
Cases of wild poliovirus types 1 (WPV1), 3 (WPV3), 1 and 3 (WPV1/WPV3), and circulating vaccine-derived poliovirus type 2 (cVDPV2) — Afghanistan, January 2012–September 2013*^†^ **Abbreviations:** FATA = Federally Administered Tribal Areas; AJK = Azad Jammu and Kashmir. * Data as of November 4, 2013. † Each dot represents one poliovirus case. Dots drawn at random within districts.

In 2012, 37 WPV1 cases were reported from 21 (5%) of 399 districts in nine (26%) of 34 provinces, compared with 80 WPV1 cases from 34 (8%) districts in 14 (41%) provinces in 2011. Of 37 WPV1 cases in 2012, 24 were reported from the Southern Region (20 cases from the 11 LPDs), six from the Eastern Region, five from the Southeastern Region, and two from the Western Region (Table, Figures 1 and 2). All nine WPV1 cases reported in 2013 to date were reported from Kunar and Nangarhar provinces in the Eastern Region; genomic sequence analysis indicates that all nine cases were caused by WPV1 originating in the bordering Federally Administered Tribal Areas (FATA) of Pakistan (Table) (2,3). Since November 2012, no WPV cases have been reported from the Southern Region. Among the 46 WPV cases reported from Afghanistan during January 2012–September 2013, 42 (91%) occurred in children aged <36 months; 16 (35%) children had not received any OPV doses through routine immunization services or SIAs, and 12 (28%) had received only 1–3 OPV doses.

From October 2012 to March 2013, 14 polio cases caused by cVDPV2 (6) were reported in the Southern Region; genomic sequence analysis indicated new emergences of cVDPV2 in 2012 as well as silent circulation of cVDPV2 lineages previously present in Afghanistan, including one lineage that had first emerged in 2009 (Table, Figures 1 and 2). The median age of children with cVDPV2 infection was 18 months, and five (36%) had never received OPV.

What is already known on this topic?

Afghanistan is one of the three remaining countries (including Pakistan and Nigeria) where indigenous wild poliovirus (WPV) transmission has never been interrupted. The Southern Region has been the main WPV reservoir area in Afghanistan.

What is added by this report?

During 2013, WPV type 1 (WPV1) transmission has declined to the lowest level since 2004. No cases of WPV1 have been reported in the Southern Region since November 2012, and WPV1 transmission in 2013 has been limited to the Eastern Region. Genomic sequence analysis indicates that all cases in 2013 were caused by WPV1 originating in the bordering Federally Administered Tribal Areas of Pakistan. WPV type 3 has not been detected since 2010. From October 2012 to March 2013, however, 14 cases of circulating vaccine-derived poliovirus type 2 were reported in the Southern Region, suggesting significant immunity gaps and raising concerns about the strength of surveillance for acute flaccid paralysis.

What are the implications for public health practice?

To achieve and maintain WPV elimination, the government of Afghanistan is improving program accountability and management capacity, strengthening surveillance, and continuing to develop and implement district level strategies to reach and vaccinate the thousands of repeatedly missed children, particularly in areas bordering Pakistan.

### Editorial Note

In 2013, WPV1 transmission in Afghanistan has declined to the lowest level since 2004. After a surge in the number of WPV cases in 2011, the government of Afghanistan and key stakeholders developed the 2012–2013 National Emergency Action Plan (NEAP) (7). Implementation of the NEAP strategies resulted in improved management and program performance and increased access to children in insecure areas. With support from the International Committee of the Red Cross and Red Crescent, negotiations with local leaders resulted in obtaining access for SIA teams into insecure areas of the Southern Region to vaccinate previously inaccessible children. Since November 2012, no WPV1 cases were reported in the Southern Region, which had been the major WPV1 reservoir area in Afghanistan. More than 3 years have passed since the last WPV3 case was reported in Afghanistan in April 2010. In addition to the observed impact on WPV transmission from 2012 to 2013, improved coverage has been indicated by the declining proportion of zero-dose NPAFP cases in children aged 6–23 months.

Staffing and training in the Southern Region LPDs were increased to improve management and accountability and to strengthen SIA planning and data management. Since early 2012, permanent polio vaccination teams comprised of local staff have worked in the Southern Region to increase OPV coverage by making quarterly visits to all households. During the first half of 2013, SIAD immunization campaigns were conducted in LPDs 1–2 weeks after SIAs to rapidly boost childhood immunity.

The magnitude of and reasons for the problem with unreached children during SIAs differed between the Southern and Eastern regions during 2012–2013. In the Southern Region, there was intermittent access to most areas, and children had opportunities to receive OPV doses. On the other hand, in some districts of the Eastern Region, children were consistently missed during successive rounds in 2013, either because of inaccessibility or poor quality SIA implementation. To provide access to OPV for children living in inaccessible areas, transit vaccination teams have been placed at border crossings into Pakistan and at the borders of inaccessible districts in the Southern and Eastern regions.

The national immunization program in Afghanistan faces major challenges because of insufficient infrastructure and financing, suboptimal cold chain equipment and procedures, low data quality for program monitoring, and lack of community engagement. Major efforts are underway to encourage development partners to work with the Ministry of Health to enhance components of the immunization program and strengthen service delivery for all vaccines in the national program. The reported cVDPV2 cases in the Southern Region indicate a major gap in poliovirus type 2 immunity because of very weak routine immunization services there; genetic sequencing of cVDPV isolates also indicate weaknesses in AFP surveillance in the past because cVDPV transmission was not detected for nearly 2 years. These weaknesses can be addressed with additional training and supervision of immunization and surveillance staff.

In August 2013, the NEAP was updated to address the current challenges faced by the program from 2013 to 2014 and to focus on those interventions that have proven effective in reaching children previously missed consistently in LPDs. Additionally, the government of Afghanistan and partners should sustain and enhance progress in vaccination team performance, community demand for vaccination, and surveillance quality, including the introduction of environmental sampling and testing. To achieve a polio-free Afghanistan, however, similar progress is needed in the remaining pockets of transmission in bordering areas of Pakistan (8).

## Figures and Tables

**TABLE:** Acute flaccid paralysis (AFP) surveillance indicators and reported cases of wild poliovirus (WPV) and circulating vaccine-derived poliovirus type 2 (cVDPV2), by region, period, and poliovirus type — Afghanistan, January 2012–September 2013*

				Reported WPV cases						
			
	AFP surveillance indicators (2012)			Period			Type		Reported cVDPV2 cases	
				
Country/Area	No. of AFP cases	Nonpolio AFP rate†	% with adequate specimens§	Jan–Jun 2012	Jul–Dec 2012	Jan–Sep 2013	WPV1	WPV3	Jul–Dec 2012	Jan–Sep 2013
**Afghanistan**	**1,829**	**9.5**	**92**	**13**	**24**	**9**	**46**	**0**	**11**	**3**
Badakhshan	53	9.8	98	0	0	0	0	0	0	0
Northeastern	233	10.9	93	0	0	0	0	0	0	0
Northern	267	11.2	94	0	0	0	0	0	0	0
Central	352	8.0	95	0	0	0	0	0	0	0
Eastern	172	8.8	94	1	5	9	15	0	0	0
Southeastern	129	6.7	93	1	4	0	5	0	0	0
Southern	315	8.9	82	10	14	0	24	0	11	3
Western	308	12.1	95	1	1	0	2	0	0	0

* Data as of November 4, 2013.

† Per 100,000 children aged <15 years.

§ Two stool specimens collected ≥24 hours apart, both within 14 days of paralysis onset, and shipped on ice or frozen packs to a World Health Organization–accredited laboratory, arriving in good condition.

## Footnotes

* The Southern Region includes Helmand, Kandahar, Zabul, Uruzgan, and Nimroz provinces; the Eastern Region includes Nangarhar, Kunar, Nuristan, and Laghman provinces.

† VDPVs can cause paralytic polio in humans and have the potential for sustained circulation of poliovirus. VDPVs resemble WPVs biologically and differ from the majority of Sabin vaccine–related poliovirus isolates by having genetic properties consistent with prolonged replication or transmission.

§ Mass campaigns conducted for a brief period (days to weeks) in which 1 dose of oral poliovirus vaccine is administered to all children aged <5 years, regardless of vaccination history. Campaigns can be conducted nationally or in portions of the country.

¶ Defined in November 2012, LPD were districts with 1) confirmed polio cases in the previous 2 years, or 2) confirmed polio cases in one of the previous 2 years, plus reported “zero-dose” nonpolio acute flaccid paralysis cases in the previous 2 years, <90% estimated oral polio vaccine coverage in the previous three SIAs, average level of community awareness of SIAs <50% in previous three SIAs, and inaccessibility in the previous three SIAs.

** SIAD campaigns are used during negotiated periods of nonviolence in otherwise inaccessible areas to vaccinate children with a monovalent OPV or bivalent OPV dose, which is administered within 1–2 weeks of the previous dose.

†† Vaccination histories of children aged 6–23 months with acute flaccid paralysis who do not test WPV-positive are used to estimate OPV coverage of the overall target population and to corroborate national reported routine vaccination coverage estimates.

§§ Areas considered too dangerous by the World Health Organization and the local government to conduct an SIA.

¶¶ The quality of AFP surveillance is monitored by performance indicators that include 1) detection rate of NPAFP cases and 2) the proportion of AFP cases with adequate stool specimens. World Health Organization (WHO) operational targets for countries with endemic poliovirus transmission are an NPAFP detection rate of at least two cases per 100,000 population aged <15 years and adequate stool specimen collection from >80% of AFP cases, in which two specimens are collected ≥24 hours apart, both within 14 days of paralysis onset, and shipped on ice or frozen packs to a WHO-accredited laboratory, arriving in good condition.
